# High serum macrophage inflammatory protein-3α is associated with the early recurrence or metastasis of non-small cell lung cancer following primary pulmonary resection

**DOI:** 10.3892/ol.2014.2229

**Published:** 2014-06-05

**Authors:** XIAOPENG ZHANG, AIHONG MENG, HUIEN WANG, XIXIN YAN

**Affiliations:** 1 Graduate School, Hebei Medical University, Shijiazhuang, Hebei, P.R. China; 2 Department of Thoracic Surgery, Hebei Province General Hospital, Shijiazhuang, Hebei, P.R. China; 3 Respiratory Division, The Second Hospital of Hebei Medical University, Shijiazhuang, Hebei, P.R. China

**Keywords:** macrophage inflammatory protein-3α, recurrence, non-small cell lung cancer, video-assisted thoracoscopic surgery, metastasis

## Abstract

The present study sought to characterize the role of macrophage inflammatory protein-3α (MIP-3α) in non-small cell lung cancer (NSCLC) patients with early recurrence or metastasis after primary pulmonary resection. Follow-up examinations were conducted for 203 NSCLC patients with primary pulmonary resection for two years post-operatively, and data was also collected for 20 healthy subjects. Serum MIP-3α levels were determined prior to surgery and at post-operative days (PODs) 30, 90 and 180, and the relevant clinical and operative variables were collected. Serum MIP-3α was measured using a commercially available enzyme-linked immunosorbent assay. There were no significant differences in age, gender and histological type among all groups (P>0.05). Serum MIP-3α levels on POD 180 were significantly higher in the recurrence group than in the non-recurrence group and healthy subjects (P=0.001). There was no significant difference in the serum MIP-3α level at PODs 90 and 180 in the patients with or without adjuvant chemotherapy (P>0.05). The recurrence rate in the high serum MIP-3α level group was 41.67%, much higher than the 23.53% observed in the low level group (P=0.006). The patients with high serum levels of MIP-3α had a significantly shorter overall recurrence-free time compared with those with low levels (P=0.004). Multivariate Cox’s regression analyses showed that only serum MIP-3α level was significant, with a hazard ratio of 1.061, a 95% confidence interval of 1.044–1.078 and a P-value of 0.001. The serum MIP-3α level in the patients with liver and bone metastases were remarkably higher than those with recurrence at other sites. The high post-operative serum MIP-3α levels were associated with an increased risk of post-operative early recurrence or metastasis in the lung cancer patients, specifically in those with bone or liver metastases.

## Introduction

Lung cancer is the second most frequently occurring cancer in the world, and is the leading cause of cancer-related mortality (1). Non-small cell lung cancer (NSCLC) has a poor prognosis, with a five-year survival rate of 40–50% in patients with pathological stage I or II disease who undergo a primary resection (2–4). Gender, age, type of surgery, tumor diameter and post-operative N-stage are significant contributing factors to the recurrence risk, with pathological stage and histological type significantly affecting recurrence-free survival (5,6)

Increased levels of inflammatory cytokines have been reported as risk factors for recurrence in a number of cancer types (7). Macrophage inflammatory protein-3 (MIP-3α), also known as CCL20, has been linked to the propagation of several malignancies, including prostate, hepatic and pancreatic carcinomas, raising the possibility that MIP-3α plays a role in lung carcinogenesis (8), thereby affecting the prognosis of lung cancer. However, there have been no studies regarding the association between the serum level of MIP-3α and the prognosis of NSCLC patients following primary resection. The present study sought to characterize the role of MIP-3α in NSCLC patients with early recurrence or metastasis.

## Materials and methods

### Study population

A total of 20 healthy subjects were enrolled, together with 239 NSCLC patients whose diagnosis (pathological Stage I or II, pre-operative assessments) was confirmed by histological examination, between February 2006 and July 2010, at the Hebei Province General Hospital (Shijiazhuang, Hebei, China). From all the patients with lung cancer who underwent pulmonary resections by video-assisted thoracoscopic surgery (VATS), the following patients were excluded: Four patients that were converted to a thoracotomy for bleeding and dense fibrous adhesions; three with pathologically-positive cancer of the bronchial stump; four that presented with surgically unresectable cancer with metastatic lymph nodes; four with pleural disseminations; five with chest wall cancer stumps; eight with serious complications or who succumbed; and eight with inadequate follow-up data. The remaining 203 patients with post-operative histopathological stage I or II NSCLC were included in this study. Staging prior to and following the surgery was based on histopathological analysis according to the International Union Against Cancer tumor-node-metastasis staging system (3). The study was approved by the Medical Ethics Committee of Hebei Province General Hospital on human research (code, 2005-123). Informed consent was obtained from each patient. Patients were followed up for two years post-operatively.

### Surgical procedures

General anesthesia with double-lumen endotracheal intubation and one-lung ventilation was used in all patients. With the VATS procedure, three working ports were made for the insertion of thoracoscopic instruments, located at the 7th or 8th intercostal space in the mid-axillary line, the 4th or the 5th intercostal space in the anterior axillary line, and the 8th or the 9th intercostal space in the scapular line. Specimen bags were used for removing the samples. Neither muscles (latissimus dorsi or serratus anterior muscles) nor ribs were cut. During the surgery, the quantity of hemorrhage (QOH) and duration of surgery (DOS) were recorded.

### Enzyme-linked immunosorbent assay (ELISA) for serum MIP-3α level

Blood samples were collected in the morning prior to the surgery and on post-operative days (PODs) 30, 90 and 180. Blood samples were also collected from healthy subjects. The serum was obtained by centrifugation at 3,000 × g for 10 min and stored at −80°C until analysis. Serum MIP-3α level was measured using a commercially available ELISA, following the manufacturer’s instrctions (RayBiotech, Inc., Norcross, GA, USA). Briefly, 96-well plates coated with the monoclonal mouse anti-human IgG1 MIP3a antibody were incubated with standards at different concentrations, and serum samples at room temperature for 2.5 h. Subsequent to three washes, the wells were incubated with a biotinylated polyclonal goat anti-human MIP3a antibody, at room temperature for 1 h, prepared HRP-conjugated streptavidin for 45 min at room temperature and TMB One-step Substrate Reagent (RayBiotech, Inc.) at room temperature for 30 min. Enzymatic reactions were developed and the absorbance was measured at 450 nm in a Multiskan FC microplate (Thermo Fisher Scientifitic, Waltham, MA, USA). Protein levels were calculated according to standard curves.

### Statistical analysis

Statistical analysis was performed using SPSS 13 (SPSS, Inc., Chicago, IL, USA). Serum MIP-3α levels and clinical and operative continuous variables are presented as the mean ± standard deviation. ANOVA and χ^2^ tests for independence were used for the comparison of clinical data and serum MIP-3α levels at each time-point. Independent sample t-tests were employed to investigate the differences in serum MIP-3α levels between patients with and without adjuvant chemotherapy. The χ^2^ test was used to examine the recurrence rate between high and low serum MIP-3α level groups. The Kaplan-Meier method was used to compare recurrence-free survival rates using a log-rank test to examine differences in recurrence free times for high and low serum MIP-3α level groups. Cox’s proportional hazards regression was further employed to estimate the hazard risk ratio of early recurrence for levels of serum MIP-3α and other clinical and operative predictors. ANOVA was used to examine differences in the serum MIP-3α levels among different metastatic or recurrent position groups. P<0.05 was considered to indicate a statistically significant difference.

## Results

### Association between tumor recurrence or metastasis and clinicopathological parameters

A total of 203 patients were reviewed for up to two years post-operatively. In total, 63 patients experienced recurrence of NSCLC, with a two-year recurrence rate of 31.0%. The serum MIP-3α levels on POD 180 were significantly higher in the recurrence group than those in the non-recurrence group and healthy subjects (P=0.001). The adjuvant chemotherapy rate was low in the non-recurrence group (P=0.035), while there were no significant differences in age, gender, QOH, DOS and histological type between the recurrence and non-recurrence groups (P>0.05) (Table I).

### Adjuvant chemotherapy has no significant effect on serum MIP-3α level

Adjuvant chemotherapy was carried out in certain patients after POD 30. To evaluate whether adjuvant chemotherapy contributes to the value of serum MIP-3α, the serum MIP-3α levels were compared at PODs 90 and 180 in the recurrence and non-recurrence groups, respectively, considering adjuvant chemotherapy as a main factor for the recurrence and non-recurrence groups. It was found that there was no significant difference in the serum MIP-3α level at PODs 90 and 180 in all groups (P>0.05) (Tables II and III).

### High serum MIP-3α level contributes to a high recurrence risk

The patients were divided into high and low serum MIP-3α level groups by the median value of the serum MIP-3α level (57 pg/ml) at POD 180. The recurrence rate in the high serum MIP-3α level group was significantly higher than that in the low serum MIP-3α level group (P=0.006) (Table IV).

### Recurrence-free survival analysis

**Figure 1:**
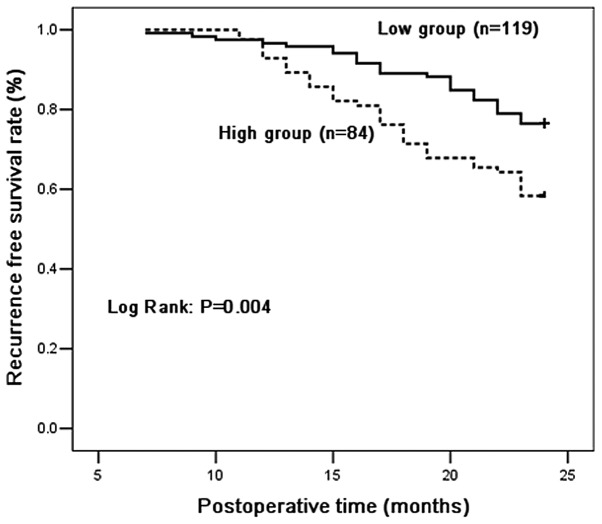
Recurrence-free survival curves for high and low serum MIP-3α groups. Patients with high serum levels of MIP-3α had a significantly shorter overall recurrence-free time compared with those with low levels (P=0.004). MIP-3α, macrophage inflammatory protein-3α.

Recurrence-free survival curves for the high and low MIP-3α groups are shown in Fig. 1. Patients in the high and low groups had two year recurrence-free rates of 76.5 and 58.3%, respectively. There were significant differences in overall recurrence-free survival between patients with high and low serum MIP-3α level. Patients with high serum levels of MIP-3α had a significantly shorter overall recurrence-free time compared with those with low levels (P=0.004).

### Multivariate Cox’s regression analysis

The study further investigated the independent effects of MIP-3α on early recurrence or metastasis with respect to age, gender, QOH, DOS, histological type and adjuvant chemotherapy rate using Cox’s regression models. Only the serum MIP-3α level on POD 180 was a significant predictor for early recurrence or metastasis. The MIP-3α level on POD 180 had a hazard ratio of 1.061, with a 95% confidence interval of 1.044–1.078 and a P-value of 0.001. In the multivariate Cox’s regression analyses, only the serum MIP-3α level was significant. Other factors were not independent predisposing factors for post-operative early recurrence or metastasis (Table V).

### Serum MIP-3α level of varying recurrence or metastasis sites

In the two years of follow-up, the earliest and average recurrence times were 7 and 17.25 months after surgery, respectively. The serum MIP-3α level was also investigated at POD 180 in the patients with varying recurrence or metastasis sites, as shown in Table VI. The serum MIP-3α levels in the patients with liver and bone metastases were significantly higher than those in the patients with other sites of recurrence (P<0.05). The patients with pleural dissemination had to be excluded from the analysis due to the small sample size.

## Discussion

MIP-3α is the only cytokine known to interact with CC chemokine receptor 6 (CCR6), a property shared with the antimicrobial β-defensins. The ligand-receptor pair MIP-3α-CCR6 is responsible for the chemoattraction of immature dendritic cells, effector/memory T cells and B cells, and plays a critical role in cancer and rheumatoid arthritis (9). MIP-3α has been linked to the development of malignant tumors. The high expression of MIP-3α has been reported in cancers of the colon (10), pancreas (11), prostate (12), nasopharynx (13) and liver (14). There is evidence that MIP-3α enhances tumor growth in numerous types of cancer (15,16). Moreover, MIP-3α is considered to be involved in carcinogenesis, angiogenesis and invasion (17,18).

Surgical procedures are an important primary approach for the treatment of early-stage lung cancer patients. However, metastasis and recurrence are the most common risks for treatment failure following surgery, with a reported 50% recurrence rate of lung cancer within one year in the treatment of NSCLC (19).

In the present study, it was found that there was a higher post-operative level of MIP-3α (POD 180) and a relatively higher adjuvant chemotherapy rate NSCLC patients (post-operative histopathological stage I or II) with recurrence or metastasis. Consistent with this finding, Kirshberg *et al* (20) reported that the MIP-3α/CCR6 axis promoted NSCLC disease progression. However, in the multivariate Cox’s regression analyses of the present study, only serum MIP-3α level (POD 180) was significant for post-operative early recurrence or metastasis, specifically for bone or liver metastasis. The study indicated that a high post-operative level of MIP-3α (POD 180) was significantly associated with the early recurrence or metastasis of NSCLC. This is the first study to identify the serum MIP-3α immune response marker as a predictor for the early post-operative recurrence of NSCLC. The study highlighted the fact that the post-operative MIP-3α level may therefore be a useful marker to determine the requirement for adjunctive anti-cancer therapy, and the fact that the MIP-3α/CCR6/IL-17 axis should be investigated further as a potential novel therapeutic target.

To the best of our knowledge, no previous study has examined the post-operative value of serum MIP-3α in NSCLC patients with adjuvant chemotherapy. In the present study, adjuvant chemotherapy was applied in certain patients after POD 30. It was found that there was no significant difference in the serum MIP-3α level at PODs 90 and 180 in all patients. This indicated that MIP-3α was independent of adjuvant chemotherapy, which was consistent with the result of the multivariate Cox’s regression analyses. Iwata *et al* (21) also showed that the serum MIP-3α status was an independent prognostic factor for overall CRC patients regardless of therapeutic interventions.

In statistical analyses, the patients of the present study were divided into low and high groups according the serum MIP-3α level following primary pulmonary resection. The patients with a high serum MIP-3α level had a higher recurrence rate than the patients with a low level. Recurrence-free survival curves showed that there were significant differences in recurrence-free survival between the two groups. This indicated that post-operative patients with high serum MIP-3α levels have a high risk of tumor recurrence or metastasis.

In further experiments, the serum MIP-3α levels were studied in patients with recurrence and different recurrence or metastasis sites. The results showed the the serum MIP-3α levels were significantly higher in the patients with liver and bone metastases. Iwata *et al* (21) also reported that the serum MIP-3α status was significantly associated with synchronous liver metastasis and was as independent predictive factor for liver metastasis.

However, several limitations, including the relatively small sample and the requirement for validation studies in independent samples, exist in this study. More studies are required to further clarify the association between MIP-3α and recurrence or metastasis in NSCLC patients.

In conclusion, in the present study, high post-operative serum MIP-3α levels were associated with an increased risk of post-operative early recurrence or metastasis in NSCLC patients (post-operative histopathological stage I or II), specifically in those with bone or liver metastases.

## Figures and Tables

**Table I:** Clinical data and serum MIP-3α level in healthy subjects, recurrence and non-recurrence groups.

Patient characteristics	Healthy	Rec (−)	Rec (+)	P-value
Total, n	20	140	63	
Age, years	62.75±9.76	62.37±8.94	62.05±9.88	0.818
Gender (male/female), n	10/10	81/59	39/24	0.587
Histological type (Ad/Sq/LC/other), n		75/61/2/2	33/27/2/1	0.930
Adjuvant/non-adjuvant chemotherapy, n		45/95	30/33	0.035
QOH, ml		99.66±28.59	94.46±26.24	0.204
DOS, min		165.89±35.19	162.77±32.16	0.537
MIP-3α, pg/ml	51.06±8.19			
Pre-operatively		48.10±12.35	51.25±10.69	0.157
POD 30		49.79±10.54	47.66±11.63	0.315
POD 90		52.83±13.66	49.74±11.55	0.268
POD 180		53.96±10.38	71.70±15.41	0.001

MIP-3α, macrophage inflammatory protein-3α; Rec (+), recurrence group; Rec (−), non-recurrence group; Ad, adenocarcinoma; Sq, squamous cell carcinoma; LC, large cell carcinoma; QOH, quantity of hemorrhage; DOS, duration of surgery; POD, post-operative day.

**Table II:** Serum MIP-3α level (pg/ml) in the recurrence group.

Day	Adjuvant chemotherapy (n=30)	Non-adjuvant chemotherapy (n=33)	P-value
POD 90	50.75±10.13	48.51±9.15	0.359
POD 180	68.94±12.11	72.49±10.23	0.192

MIP-3α, macrophage inflammatory protein-3α; POD, post-operative day.

**Table III:** Serum MIP-3α level (pg/ml) in the non-recurrence group.

Day	Adjuvant chemotherapy (n=45)	Non-adjuvant chemotherapy (n=95)	P-value
POD 90	53.31±11.12	49.59±11.75	0.077
POD 180	55.83±9.81	52.59±10.75	0.089

MIP-3α, macrophage inflammatory protein-3α; POD, post-operative day.

**Table IV:** Recurrence rate in the high and low serum MIP-3α level groups.

serum MIP-3α level at POD 180, pg/ml	Number	Rec (−)	Rec (+)	Rate, %
Low (<57), n	119	91	28	23.53
High (>57), n	84	49	35	41.67

P=0.006. MIP-3α, macrophage inflammatory protein-3α; Rec (+), recurrence group; Rec (−), non-recurrence group; POD, post-operative day.

**Table V:** Recurrence factors in Cox’s proportional hazards model.

Patient characteristics	HR (95% CI)	P-value
Age, years	0.989 (0.962–1.017)	0.443
Gender (male/female)	0.990 (0.544–1.803)	0.974
Histological type (Ad/Sq/LC/Other)	0.508 (0.989–2.298)	0.076
Adjuvant/non-adjuvant chemotherapy	1.405 (0.976–1.985)	0.069
QOH, ml	0.996 (0.987–1.135)	0.407
DOS, min	1.002 (0.994–1.010)	0.624
MIP-3α, pg/ml		
Pre-operatively	1.003 (0.985–1.021)	0.730
POD 30	1.012 (0.986–1.038)	0.366
POD 90	1.002 (0.984–1.020)	0.821
POD 180	1.061 (1.044–1.078)	0.001

HR, hazard ratio; CI, confidence interval; Ad, adenocarcinoma; Sq, squamous cell carcinoma; LC, large cell carcinoma; QOH, quantity of hemorrhage; DOS, duration of surgery; MIP-3α, macrophage inflammatory protein-3α; POD, post-operative day.

**Table VI:** Post-operative tumor recurrence or metastasis site and serum MIP-3α level.

Recurrence site	Number (n=63)	Serum MIP-3α level at POD 180, pg/ml
Lung	10	65.92±9.15
Lymph node	11	64.45±8.49
Brain	7	67.65±8.24
Bone	7	85.10±9.95^a^
Liver	6	87.31±11.12^a^
Pleural dissemination	2	68.85±2.33
Lung+mediastinal lymph node	7	67.95±8.40
Lung+brain	7	66.67±9.74
Lung+bone	6	88.34±12.46^a^

a P<0.05 vs. other recurrence sites.

The patients with pleural dissemination were excluded in analysis due to the small sample size. MIP-3α, macrophage inflammatory protein-3α; POD, post-operative day.
